# Male hypogonadism in patients on maintenance hemodiafiltration: prevalence and therapeutic effect

**DOI:** 10.3389/fendo.2026.1744953

**Published:** 2026-01-28

**Authors:** Marcia S. Carvalho, Raissa C. Torres, Isabela M. Toniasso, Andrea O. Magalhães, Cynthia M. Borges, Erica P. Rocha, Christiane A. Kojima, Rosilene M. Elias

**Affiliations:** 1 Nefrostar Kidney Care, Sao Paulo, Brazil; 2 Splendore Kidney Care, Sao Paulo, Brazil; 3 Faculdade São Leopoldo Mandic Campinas, São Paulo, Brazil; 4 Department of Medicine, Service of Nephrology, University of Sao Paulo, Sao Paulo, Brazil; 5 Universidade Nove de Julho, Sao Paulo, Brazil

**Keywords:** chronic kidney disease, hemodiafiltration, hypogonadism, kidney replacement therapy, testosterone

## Abstract

**Aims:**

Male hypogonadism is highly prevalent among patients undergoing dialysis and carries significant clinical implications, although is often overlooked. Testosterone deficiency in this population is associated with adverse clinical outcomes, mainly because of cardiovascular disease, protein energy wasting and infection complication. Therefore, identify these patients is a high clinical priority.

**Methods:**

This is a cross-sectional study that enrolled adult men on maintenance hemodiafiltration in four dialysis centers. Hypogonadism was defined by two consecutive measurements of serum total testosterone levels. Free testosterone was calculated based on serum albumin and sex hormone-binding globulin. Symptoms of androgen deficiency were evaluated using androgen deficiency in aging males (ADAM) questionnaire. The presence of comorbidities and laboratory markers was also evaluated.

**Results:**

Hypogonadism was identified in 59 out of 121 patients (48.7%). Patients with hypogonadism were older (62 ± 15 vs. 57 ± 15 years, *p* < 0.001) and had higher prolactin levels (22 [13–36] vs. 14 [10–18] ng/mL, *p* = 0.002). No other significant difference was observed in demographic, clinical, or laboratory features between patients with and without hypogonadism. Among the 16 patients who received testosterone supplementation, 66.7% showed improvement in ADAM scores, with the median score decreasing from 3 (2–4) to 1 (0–2) (*p* = 0.003).

**Conclusion:**

More than one-third of men undergoing hemodiafiltration were diagnosed with hypogonadism. Aside from older age, no other distinguishing characteristics were identified in this population. Therefore, routine assessment of testosterone levels should be considered for all men undergoing dialysis. Further studies are needed to determine whether hormone supplementation can improve clinical outcomes.

## Introduction

Male hypogonadism is a highly prevalent disorder among patients with chronic kidney disease (CKD), ranging from 26% to 70% that has a significant impact on patient health (1–4).

Low testosterone levels have been consistently associated with increased cardiovascular risk and poorer clinical outcomes in patients with CKD on dialysis. In this context, testosterone deficiency appears to function primarily as a biological marker of systemic illness and frailty, reflecting underlying processes such as chronic inflammation, endothelial dysfunction, insulin resistance, altered lipid metabolism, arterial stiffness, sarcopenia and reduced functional capacity, rather than acting as an isolated causal factor. These interrelated abnormalities collectively contribute to higher morbidity and mortality in this population (5–8).

Given this complex scenario, early detection and appropriate management of hypogonadism are essential to improve quality of life and reduce complications (9). Early diagnosis, coupled with an individualized and multidisciplinary management approach, is therefore crucial.

Data on male hypogonadism and testosterone supplementation in dialysis-dependent patients remain limited, largely derived from small observational cohorts and single-center studies (3, 23, 29). Hemodiafiltration is a technique associated with better outcomes and has been recognized as superior to hemodialysis worldwide (10–14). The prevalence of hypogonadism in patients undergoing hemodiafiltration is unknown. In this study, we aimed to assess the prevalence of hypogonadism among men with CKD undergoing maintenance hemodiafiltration, and to discuss the diagnostic challenges and treatment response to testosterone supplementation.

## Materials and methods

This is a cross-sectional observational study that enrolled men aged ≥18 years undergoing maintenance hemodiafiltration in four dialysis centers in São Paulo, Brazil. The age range observed in the cohort was 18 to 89 years, with patients categorized into three age strata (≤ 60, 61–70, ≥ 71 years) as detailed in **Table 1**.

**Table 1:** Characteristics of patients according to the presence of hypogonadism.

Variable	All	Without hypogonadism	With hypogonadism	p
	(n=121)	(n=62)	(n=59)	
Age, y	57 ± 15	52 ± 13	62 ± 15	<0.001
Age group				<0.001
Until 60 y	70 (57.9)	44 (71.0)	26 (44.1)	
61–70 y	25 (20.7)	13 (21.0)	12 (20.3)	
≥ 71 y	26 (21.5)	5 (8.1)	21 (35.6)	
BMI, kg/m^2^	27.0 ± 5.0	26.7 ± 5.1	27.2 ± 5.0	0.554
BMI classification				0.534
Normal, %		26 (41.3)	20 (34.5)	
Overweight, %		21 (33.3)	18 (31.0)	
Obese, %		16 (25.4)	20 (34.5)	
Time on dialysis, m	23 (14–36)	23 (12–36)	23 (14–33)	0.988
Diabetes, n (%)	54	27 (42.9)	27 (46.6)	0.683
Hypertension, n (%)	81	45 (71.4)	36 (62.1)	0.274
CVD, n (%)	36	18 (28.6)	18 (31.0)	0.767
Smoking habits, n (%)	30	15 (24.2)	15 (25.9)	0.833
Hemoglobin, g/dL	11.6 ± 1.4	11.5 ± 1.4	11.7 ± 1.4	0.431
Glucose	121.1 ± 58.9	118.2 ± 51.9	124.3 ± 62.0	0.562
Hemoglobin A1c, %	6.1 ± 1.5	6.1 ± 1.5	6.1 ± 1.4	0.983
Albumin, g/dL	3.8 ± 0.3	3.8 ± 0.3	3.8 ± 0.3	0.487
25(OH) vitamin D, ng/	28.0 ± 11.4	27.6 ± 12.5	28.5 ± 10.2	0.661
TSH, µU/mL	1.7 (1.1-2.7)	1.7 (1.2-2.5)	1.7 (1.0-2.8)	0.889
Free T4, ng/dL	0.75 (0.64-0.92)	0.76 (0.64-0.91)	0.74 (0.65-0.95)	0.932
FSH, mlU/mL	7.8 (4.3-14.1)	7.2 (3.6-11.8)	8.5 (5.1-21.4)	0.039
LH, IU/L	8.6 (5.5-12.8)	7.5 (5.1-10.7)	9.6 (5.9-17.5)	0.050
Prolactin, ng/mL	16.4 (11.4-26.1)	14.1 (10.0-18.3)	22.2 (13.2-36.3)	0.002
Calculated testosterone, ng/dL	6.7 ± 3.5	8.7 ± 3.4	4.6 ± 2.0	<0.001
SHBG, nmol/L	37.1 (24.6-51.9)	34.9 (23.5-48.0)	41.0 (27.3-58.7)	0.313

BMI, body mass index; CVD, cardiovascular disease; TSH, thyroid stimulating hormone; FSH, follicle-stimulating hormone; LH, Luteinizing Hormone; SHBG, Sex Hormone-Binding Globulin.

The primary outcome was the prevalence of hypogonadism, determined by two consecutive measurements of serum total testosterone levels. Free testosterone was calculated based on serum albumin and sex hormone-binding globulin (SHBG), using a validated equation Free & Bioavailable Testosterone calculator, as originally described by Vermeulen et al. (1999), through the validated calculator developed at the University Hospital of Ghent, Belgium. These calculated parameters more accurately reflect the level of bioactive testosterone than does the sole measurement of total serum testosterone.

Testosterone and dihydrotestosterone circulate in plasma unbound (free approximately 2 - 3%), bound to specific plasma proteins (sex hormone-binding globulin SHBG) and weakly bound to nonspecific proteins such as albumin. The SHBG-bound fraction is biologically inactive because of the high binding affinity of SHBG for testosterone. Free testosterone measures the free fraction; bioavailable testosterone includes free plus weakly bound to albumin. This calculator was developed at the Hormonology department, University Hospital of Ghent, Belgium.

Hormonal measurements were done using the chemiluminescence method, considering reference values for men of total testosterone between 175 and 781 ng/dL and of SHBG for men aged 20 to 50 years between 13.3 and 89.5 nmol/L. The cutoff value for calculated testosterone was considered > 6.5 ng/dL (15–17). The diagnosis of hypogonadism was based on total morning serum testosterone levels < 264 ng/dL (9.2 nmol/L), in accordance with the Endocrine Society Clinical Practice Guideline (19). Blood samples were obtained in the morning (between 7:00 and 10:00 AM) due to circadian variation, and the diagnosis was confirmed by a second measurement performed at least 30 days after the initial abnormal result (10, 25).

Symptoms of androgen deficiency was tested using questionnaire for androgen deficiency in aging males (ADAM) (18).

The presence of comorbidities and relevant laboratory markers was also evaluated. To ascertain the diagnose of secondary hypogonadism, sexual hormone (follicle-stimulating hormone - FSH and luteinizing hormone- LH) and prolactin were evaluated. Clinical and demographic data included age, sex, body mass index, smoking habits, and history of diabetes, cardiovascular disease and hypertension. Laboratory data recorded from charts included hemoglobin (analysis performed by fluorescent flow cytometry and impedance, reference value for adult men from 13 to 16.5 gd/L and men over 70 years of age from 11.5 to 15.1 g/dL), glucose (analysis performed by enzymatic method - hexokinase, reference value for adults from 74 to 104 ng/dL), hemoglobin A1C (analysis by the turbidimetric inhibition immunoassay method, reference value from 5.7 to 6.4%), albumin (analysis by the bromocresol green method, reference value from 3.2 to 4.8 g/dL), thyroid-stimulating hormone - TSH (analysis by the chemiluminescence method, reference value for adults from 0.55 to 4.78 µIU/ml), free T4 (analysis by the chemiluminescence method, reference value for adults from 0.89 to 1.76 ng/dL), FSH analysis by chemiluminescence method, reference value for men from 1.27 to 19.26 mIU/mL), LH (analysis by chemiluminescence method, reference value for men from 1.24 to 8.62 mIU/mL) and prolactin (analysis by chemiluminescence method, reference value for men from 2.64 to 13.13 ng/dL).

Testosterone replacement therapy consisted of either intramuscular testosterone undecanoate administered at a dose of 1000mg every three months or daily transdermal testosterone gel (16.2 mg/g per pump twice a day). Post-replacement testosterone levels were measured before gel or intramuscular (IM) administration of the subsequent dose, 3 months later for patients receiving transdermal therapy and 6 months later for those receiving IM therapy. Patients were screened in the period between January 1^st^ 2024 and 31^st^ December 2024. Laboratory data was considered within one month of the study inclusion. ADAM questionary was applied at the baseline and 3 months after hormone supplementation. Reasons that contraindicated this therapy were recorded.

Inclusion criteria were adult men on maintenance hemodiafiltration in the 4 dialysis centers included in the study. any dialysis unit. The same endocrinologist collected the clinical and demographic data and applied the questionnaire. Exclusion criteria were hospitalization and current treatment of infection, known pituitary or hypothalamic disease, prior diagnosis of primary or secondary hypogonadism, current or recent use of exogenous androgens, anabolic steroids or systemic corticosteroids, active malignancy, and other severe endocrine disorders apart from controlled diabetes mellitus and thyroid dysfunction.

All patients included signed the written informed consent approved by the Ethic Board Committee Regional Society of Education and Health LTDA (CAAE = 82067824.3.0000.5374/opinion number 7,081,213).

Sample size was obtained by convenience.

### Data analysis

Descriptive statistics were expressed as mean ± standard deviation (SD) or median (interquartile range, IQR) for continuous variables, and as frequencies and percentages for categorical variables. Normality of continuous data was assessed using the Shapiro-Wilk test.

Comparisons between patients with and without hypogonadism were performed using the independent samples *t*-test for normally distributed continuous variables, or the Mann–Whitney *U* test for non-normally distributed variables. Categorical variables were compared using the chi-square test or Fisher’s exact test, as appropriate. Binary regression analysis was performed to evaluated independent factors associated with hypogonadism.

Data were analyzed using SPSS™ version 29 (SPSS Inc., Chicago, IL, United States of America) and Graphpad Prism™ version 10 (GraphPad Software, La Jolla, CA, United States of America). A two-sided *p* value <0.05 was considered statistically significant.

## Results

**Figure 1:**
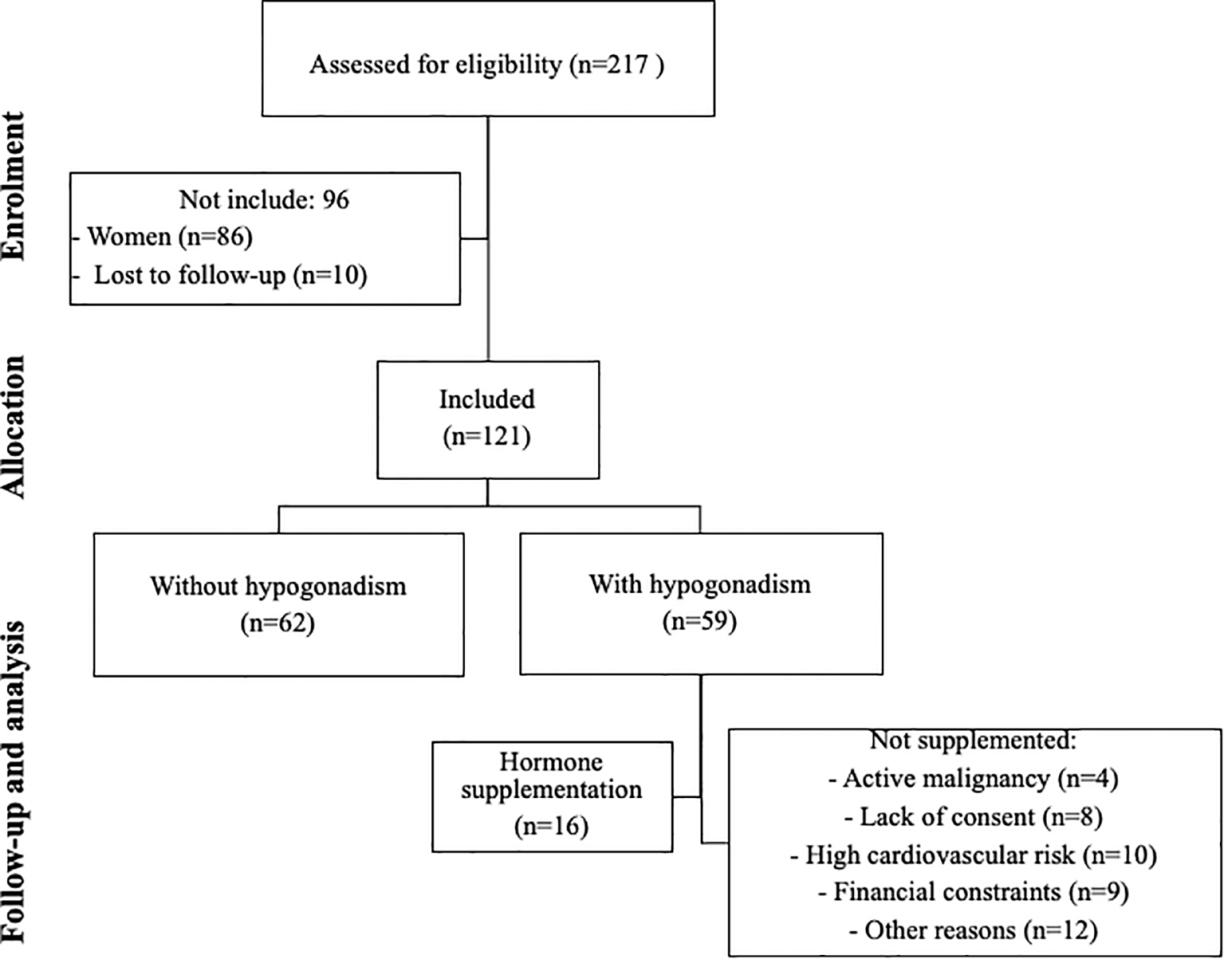
Flowchart of study enrollment.

All patients in the four dialysis centers were screened for inclusion criteria. After the initial selection, 10 patients were transferred to another dialysis unit, and 121 men were included in the final analysis. Hypogonadism was identified in 59 patients (48.7%), and 16 of them received supplementation after evaluation for usual contraindication (**Figure 1**). Characteristics of patients according to the presence of hypogonadism is found in **Table 1**. Patients with hypogonadism were older (particularly in those ≥ 71 years old), had higher FSH, LH, and prolactin levels. No other significant differences were observed in demographic, clinical, or laboratory characteristics between patients with and without hypogonadism. Binary regression analysis showed that age (HR 1.050 95% CI 1.020-1.080, p<0.001), but not prolactin (p=0.180), was significantly associated with the risk of hypogonadism. Calculated testosterone had an inverse correlation with FSH (r=-0.345, p<0.01), LH (r=-0.290, p=0.003), prolactin (r=-0.288, p=0.002) and age (r=-0.405, p<0.001).

**Figure 2:**
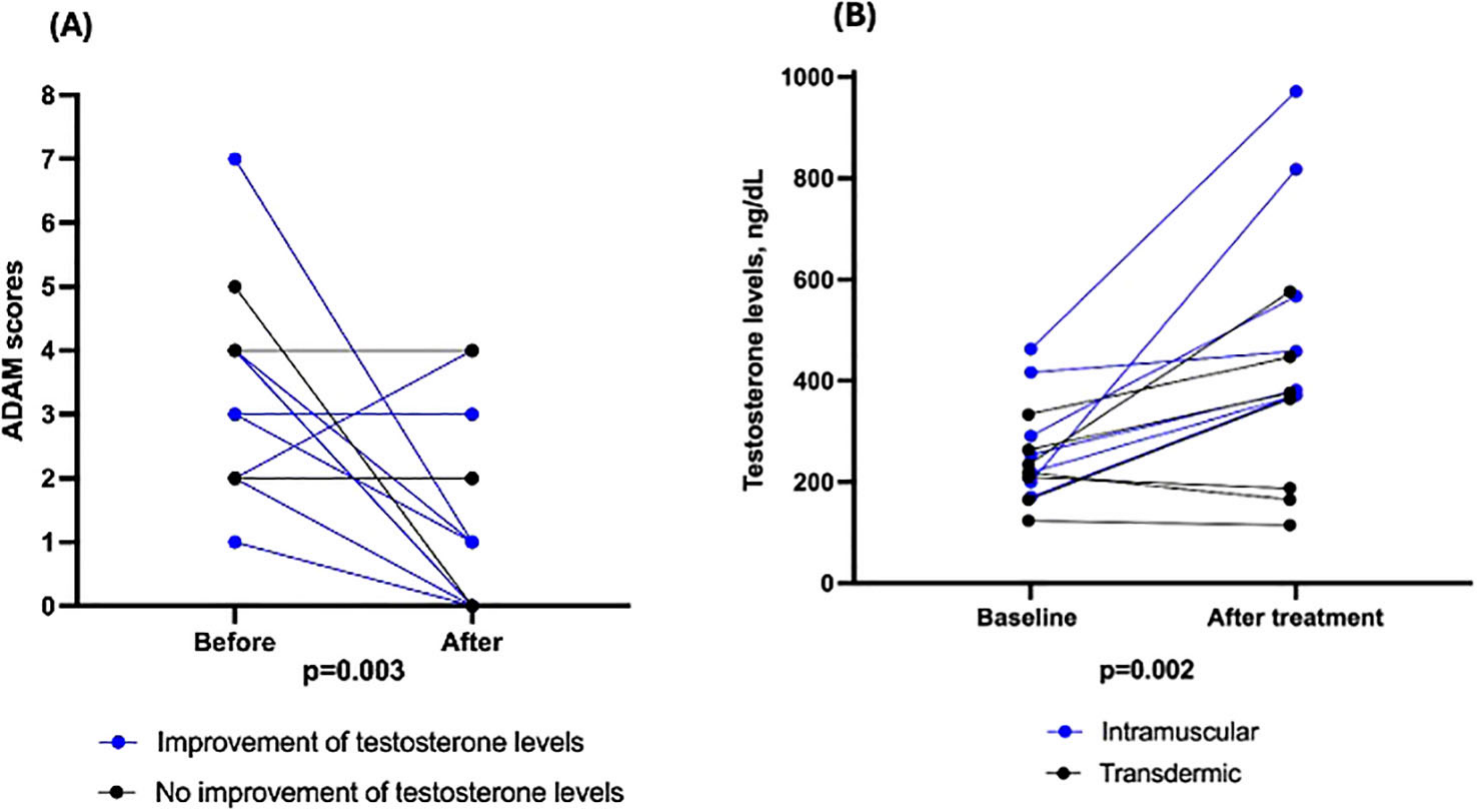
**(A)** Score obtained from the Androgen Deficiency in Aging Males (ADAM) questionnaire before and after testosterone supplementation. Blue and black lines and symbols represent patients with and without concomitant increase in testosterone levels, respectively. **(B)** Levels of testosterone before and after supplementation, according to administration route (blue for intramuscular and black for transdermic).

Among 16 patients who received testosterone supplementation, 68.7% exhibited a significant improvement in ADAM scores, with the median score decreasing from 3 (2–4) at baseline to 1 (0–2) after treatment (p = 0.003) (**Figure 2A**). In general, ADAM scores improved in patients who experienced an increase in testosterone levels. However, one patient showed an improvement in ADAM scores without a concomitant increase in testosterone levels, and another patient exhibited an increase in testosterone levels without a corresponding improvement in ADAM scores (**Supplementary Table 1**). Notably, biochemical normalization of serum testosterone levels was achieved in 11 patients (68.7%), with median levels increasing from 242.5 ng/dL to 376 ng/dL (p = 0.002), irrespective of the route of administration, as shown in **Figure 2B**.

## Discussion

In this study we investigated the prevalence of male hypogonadism in patients on maintenance hemodiafiltration. The major findings are that: 1) nearly half of patients presented with hypogonadism, 2) those with hypogonadism were older and had higher prolactin levels, and 3) testosterone supplementation appeared to improve symptoms, as reflected by ADAM scores.

Significant and frequent endocrine alterations occur in patients with CKD, including hyperprolactinemia, insulin resistance, deficiencies of reproductive and thyroid hormones. These hormonal disorders contribute to increased morbidity and reduced quality of life. Male hypogonadism is observed across all stages of CKD, involving patients on conservative management, dialysis, and kidney transplant recipients (19). The mechanisms underlying the association between testosterone deficiency and kidney disease are multifactorial and include chronic inflammation and malnutrition (20), anemia, and reduced responsiveness to erythropoietin caused by testosterone deficiency (21). In our study, we identified a high prevalence of male hypogonadism in this study (48.7%), consistent with previous reports in patients with CKD undergoing dialysis (3, 22, 23).

A major challenge in evaluating male hypogonadism is establishing the diagnosis, which requires unequivocally and consistently low serum testosterone concentrations. When SHBG is abnormal, free testosterone should be calculated. The age-related decline in testosterone (usually 1% per year) is more pronounced in free testosterone due to the increase in SHBG levels that occurs with advancing age and in CKD progression (24, 25). Elevated SHBG reduces the levels of free circulating testosterone, which is a biologically active form of the hormone (26). In the current study, we considered total morning testosterone levels below 264 ng/dL (9.2 nmol/L) as the diagnostic threshold, in accordance with the recommendations of the Endocrine Society (16). This recommendation is based on the natural diurnal variation of testosterone, which typically peaks in the morning (27, 28). All samples were analyzed using a high-quality validated laboratory method. In addition, serum testosterone levels in our patients were confirmed by a second measurement performed at least 30 days apart when the first result was below 264 ng/dL (16, 19).

Clinical evaluation, in addition to biochemical testing (27, 28), is essential for the evaluation of male hypogonadism. However, the signs and symptoms of hypogonadism often overlap with those observed in patients on dialysis, making laboratory evaluation indispensable. Typical manifestations included reduced libido, erectile dysfunction, fatigue, loss of muscle mass and strength, increased body fat, decreased bone density, and mood disturbances. he clinical approach should also include a thorough assessment of potential contributing factors, such as chronic comorbidities, medication use, and lifestyle habits that may influence testosterone levels.

In our study, we performed a comprehensive evaluation to exclude other conditions that might mimic or exacerbate testosterone deficiency, including measurements of prolactin, thyroid function, and iron stores, and pituitary imaging when indicated (2, 16). Although the ADAM questionnaire is a sensitive screening tool for androgen deficiency, its specificity is limited in patients with advanced CKD, as several uremia-related symptoms (fatigue, reduced energy, depressive mood, and decreased physical performance) overlap with manifestations of hypogonadism. To minimize misclassification, the clinical interpretation of ADAM in the present study placed primary emphasis on the sexual domains of the instrument, particularly decreased libido (item 1) and erectile dysfunction (item 7), which were considered the main drivers of the clinical suspicion of hypogonadism and subsequent diagnostic confirmation. Non-sexual items were interpreted cautiously and in conjunction with biochemical testosterone measurements and the overall clinical context. The use of ADAM scores proved useful in identifying symptoms that might otherwise be overlooked or misattributed to aging or uremia-related complaints. Among patients who received testosterone supplementation, 66.7% reported an improvement of symptoms. A pilot randomized study enrolling patient on hemodialysis found 6 of 11 patients with hypogonadism, with an improvement of sexual function after treatment (29). Unfortunately, a considerable proportion of patients were not eligible for treatment due to classical contraindications frequently encountered in individuals with CKD on hemodialysis. Another small, randomized studies (6 and 13 patients treated) (23, 29), showed improvement of symptoms after testosterone supplementation.

The importance of the diagnose relies on the impact of hypogonadism in adverse outcomes. A meta-analysis involving 28,663 men with CKD and controls found that those with the lowest testosterone levels had significantly higher mortality rates, highlighting the potential impact of testosterone deficiency on patient outcomes (30). In a *post-hoc* analysis of the Evaluation of Cinacalcet Therapy of Lower Cardiovascular Events (EVOLVE) randomized controlled trial, low testosterone was identified as a biomarker of morbidity and mortality in advanced kidney disease. Among hemodialysis patients, low testosterone levels and elevated levels of SHBG in men on hemodialysis were associated to an increased cardiovascular morbidity and mortality (31, 32).

In patients with CKD on hemodialysis, low testosterone levels have been associated with deteriorated surrogate markers, of quality of life (33). Several mechanisms mat explain this relationship. Testosterone plays a crucial role in muscle mass, bone density, erythropoiesis, reduction of inflammatory substances, especially among patients with diabetes. Bone disease, often observed in this population, can be exacerbated by testosterone deficiency and thus influences morbidity and potentially mortality (28, 34–39).

Testosterone replacement therapy in patients with CKD requires careful risk–benefit assessment. Potential adverse effects include exacerbation of polycythemia with increased hematocrit, worsening of obstructive sleep apnea, fluid retention, and potential cardiovascular risks in an already high-risk population. In our cohort, 42 patients with hypogonadism were not treated due to classical contraindications or clinical considerations, including active malignancy (n=4), high cardiovascular risk (n=10), treatment refusal (n=8), high cost (n=9), and other medical reasons (n=11), as depicted in **Figure 1**.

Our study has several limitations, including a short follow-up period; the cross-sectional design, which precludes causal inference; a small treatment sample size (n = 16); the absence of randomization or a control group for supplementation; potential selection bias due to the use of a convenience sample; the large number of patients who were not eligible for treatment; and the lack of data on additional outcomes, such as quality of life, anemia, and muscle mass. Moreover, these findings may not be generalizable to other patients undergoing hemodiafiltration, including those in Brazil.

However, the aim of the study was specifically to evaluate the prevalence of hypogonadism and the management of androgen-related symptoms with treatment, which was achieved. A further strength was the rigorous diagnostic approach process and the inclusion of a relatively large cohort of patients on dialysis, compared with previous studies in this population.

In conclusion, male hypogonadism is highly prevalent among patients on dialysis, and symptoms may improve with testosterone supplementation when guided by careful clinical evaluation and a balanced assessment of risks and benefits.

## Data Availability

The raw data supporting the conclusions of this article will be made available by the authors, without undue reservation.
