# Supplementary material for: Male hypogonadism in patients on maintenance hemodiafiltration: prevalence and therapeutic effect
Source: Front Endocrinol (Lausanne). 2026 Jan 28;17:1744953. doi: 10.3389/fendo.2026.1744953 (PMC12890636; doi:10.3389/fendo.2026.1744953)
Supplement: Supplementary file 1 [file Table1.docx]

**Supplementary Table 1.**

| **Patient number** | **Administration via** | **ADAM scores improvement** | **Baseline testosterone levels (ng/mL)** | **Post replacement testosterone levels (ng/mL)** | **Change (%)** |
| --- | --- | --- | --- | --- | --- |
| 1 | IM | Yes | 287 | 558.93 | 94.75 |
| 2 | Transdermic | No | 124 | 115 | -7.26 |
| 3 | Transdermic | Yes | 235 | 575.9 | 145.06 |
| 4 | IM | Yes | 255 | - |  |
| 5 | Transdermic | No | 209 | 188 | -10.05 |
| 6 | Transdermic | Yes | 333 | 447 | 34.23 |
| 7 | IM | Yes | 168 | 366 | 117.86 |
| 8 | Transdermic | Yes | 218 | 165 | -24.31 |
| 9 | IM | Yes | 456 | 958 | 110.08 |
| 10 | Transdermic | Yes | 440 | - |  |
| 11 | IM | No | 216 | 366 | 69.44 |
| 12 | IM | Yes | 250 | 376 | 50.40 |
| 13 | Transdermic | No | 165 | 364 | 120.61 |
| 14 | Transdermic | Yes | 263 | 376 | 42.97 |
| 15 | IM | No | 197 | 806 | 309.14 |
| 16 | IM | Yes | 411 | 452 | 9.97 |
